# Safety and practicability of using mid-upper arm circumference as a discharge criterion in community based management of severe acute malnutrition in children aged 6 to 59 months programmes

**DOI:** 10.1186/s13690-016-0136-x

**Published:** 2016-06-15

**Authors:** Paul J. Binns, Nancy M. Dale, Theresa Banda, Chrissy Banda, Bina Shaba, Mark Myatt

**Affiliations:** Valid International, Oxford, UK; Department for International Health, University of Tampere, Tampere, Finland; Valid International, Lilongwe, Malawi; Valid International, Lilongwe, Malawi; Valid International, Lilongwe, Malawi; Brixton Health, Llawryglyn, Wales UK

**Keywords:** Severe Acute Malnutrition (SAM), Mid Upper Arm Circumference (MUAC), Discharge criteria

## Abstract

**Background:**

The use of proportional weight gain as a discharge criterion for MUAC admissions to programs treating severe acute malnutrition (SAM) is no longer recommended by WHO. The critical limitation with the proportional weight gain criterion was that children who are most severely malnourished tended to receive shorter treatment compared to less severely malnourished children. Studies have shown that using a discharge criterion of MUAC ≥ 125 mm eliminates this problem but concerns remain over the duration of treatment required to reach this criterion and whether this discharge criterion is safe. This study assessed the safety and practicability of using MUAC ≥ 125 mm as a discharge criterion for community based management of SAM in children aged 6 to 59 months.

**Methods:**

A standards-based trial was undertaken in health facilities for the outpatient treatment of SAM in Lilongwe District, Malawi. 258 children aged 6 to 51 months were enrolled with uncomplicated SAM as defined by a MUAC equal or less than 115 mm without serious medical complications. Children were discharged from treatment as ‘cured’ when they achieved a MUAC of 125 mm or greater for two consecutive visits. After discharge, children were followed-up at home every two weeks for three months.

**Results:**

This study confirms that a MUAC discharge criterion of 125 mm or greater is a safe discharge criterion and is associated with low levels of relapse to SAM (1.9 %) and mortality (1.3 %) with long durations of treatment seen only in the most severe SAM cases. The proportion of children experiencing a negative outcome was 3.2 % and significantly below the 10 % standard (*p =* 0.0013) established for the study. All children with negative outcomes had achieved weight-for-height z-score (WHZ) above −1 z-scores at discharge. Children admitted with lower MUAC had higher proportional weight gains (*p <* 0.001) and longer lengths of stay (*p <* 0.0001). MUAC at admission and attendance were both independently associated with cure (*p <* 0.0001). There was no association with negative outcomes at three months post discharge for children with heights at admission below 65 cm than for taller children (*p =* 0.5798).

**Conclusions:**

These results are consistent with MUAC ≥ 125 mm for two consecutive visits being a safe and practicable discharge criterion. Use of a MUAC threshold of 125 mm for discharge achieves reasonable lengths of stay and was also found to be appropriate for children aged six months or older who are less than 65 cm in height at admission. Early detection and recruitment of SAM cases using MUAC in the community and compliance with the CMAM treatment protocols should reduce lengths of stay and associated treatment costs.

## Background

The use of mid-upper arm circumference (MUAC) as a *general* tool (i.e. for case-finding, referral, admission, monitoring response to treatment, and discharge) has the potential to simplify the practical application of community-based management of acute malnutrition (CMAM) services, facilitating their delivery within the framework of primary and community health services.

In 2007 the use of MUAC for case-finding, referral, and admission to CMAM services was accepted by the World Health Organisation (WHO), the World Food Program (WFP), the United Nations Standing Committee on Nutrition (UN/SCN), and the United Nations Children’s Fund (UNICEF) [1]. MUAC is now widely used in CMAM programs for these purposes.

The use of proportional weight gain as a discharge criterion for MUAC admissions is no longer recommended by WHO [2], as it had proven to be problematic with the most malnourished cases receiving the least amount of treatment. While the use of MUAC greater than 125 mm has been used in some settings prior to the WHO updated recommendations and some studies have proposed MUAC as an alternative discharge criterion to percentage weight gain [3, 4] for MUAC admissions, the safety of using MUAC greater than 125 mm as a discharge criterion has not been validated [2].

A consultation between academics and non-governmental organisations (NGOs) in December 2012 [5] concluded that there were concerns over the safety of using MUAC as a discharge criterion due to a lack of evidence with regard to post-discharge relapse and mortality rates. Also, anecdotal evidence suggested that children over six months of age with heights less than 65 cm admitted with low MUAC would experience very long lengths of stay and may not reach the MUAC discharge threshold within sixteen weeks (112 days) of admission. Published evidence was considered to be inadequate to inform programming.

The aims of this study were to evaluate the safety and practicability of using MUAC as a discharge criterion for children aged 6 to 59 months admitted to outpatient treatment for SAM using MUAC.

### Ethics statement

Written consent was obtained from a parent (or primary caregiver) of the child to be enrolled in the study. Each parent/caregiver signed an individual consent form detailing the proposed treatment, the right to refuse enrolment into the study, the right to confidentiality and anonymity and that no payment would be received for participation.

This study complied with World Medical Association Declaration of Helsinki (Ethical Principles for Medical Research Involving Human Subjects, 1964) and was approved by the National Health Sciences Research Committee of Malawi (NHSRC, protocol # 817).

## Methods

The study design was a standards-based trial. There are no internationally agreed standards for post-discharge relapse and mortality in CMAM programs. The standard adopted for the study was informed by a review by Ashworth [6, 7], which used a standard of < 10 % relapse as a ‘criterion for success’ for community-based nutritional rehabilitation programmes prior to the development of the CMAM treatment approach. A standard of less than 10 % for combined post discharge relapse and non-accidental, non-violent death was adopted for the study through consultation with experts in CMAM programming at FANTA/AED. This standard was more stringent than the standard used by Ashworth. In this article we present estimates for post-discharge relapse and mortality separately and combined so as to allow comparison against other standards.

The study was conducted in five outpatient health facilities run by the Government of Malawi Ministry of Health (MoH) and located in Lilongwe District, Malawi, with care delivered by MoH staff. A research team from Valid International (comprised of a research coordinator, a registered nurse, and a community mobilisation officer) trained MoH staff in study protocols and assisted directly in the measurement and treatment of all study subjects. The Malawi National Guidelines for Community-based Therapeutic Care [8] formed the standard of care. At the start of the study the admission criterion for the treatment of SAM was MUAC < 110 mm according to the Malawi National guidelines. These guidelines were in the process of being revised to meet the recommendation of the UN Joint Statement of 2009 [2] to raise the admission criteria to MUAC < 115 mm. The clinics in which the study subjects were recruited changed the admission criteria to MUAC ≤ 115 mm ahead of the publication of the revised guidelines with the consent of the Ministry of Health.

The study enrolled children aged between 6 and 59 months with uncomplicated severe acute malnutrition (SAM) as defined by MUAC ≤ 115 mm and without medical complications. Children with a MUAC ≤ 115 mm with concurrent bilateral pitting oedema or medical complications were referred to inpatient care according to Malawi National Guidelines and enrolled into the study on their return from inpatient care.

Subjects were recruited between February 2011 and March 2012. Age was verified by reference to the child’s ‘health passport’. Children whose caregivers were resident outside of Lilongwe District were excluded from the study but were admitted to the CMAM program and provided with standard care according to the Malawi National Guidelines. MUAC, weight and height were measured on admission. An appetite test was conducted to confirm the appropriate consumption of Ready to Use Therapeutic Food (RUTF) which is required in order to receive treatment as an outpatient.

Routine medications given on admission followed the Malawi National Guidelines and included a broad spectrum antibiotic (amoxicillin or co-trimoxazole) and vitamin A. Each child was assessed for malaria using a rapid diagnostic test, (Paracheck Pf®), and if positive, was treated with antimalarials (lumefantrine and artemether). If the Paracheck Pf®, rapid test was unavailable, the patient was treated empirically for malaria if they presented with a fever of unknown origin. Each caregiver and child was routinely offered serial testing for Human Immunodeficiency Virus (HIV) using the Determine HIV-1/2 Assay™ and, if tested positive, Uni-Gold Recombigen HIV assay™. HIV positive cases were included in the study and referred for appropriate treatment. It was not monitored whether the child went on to receive treatment for HIV. At each visit the child was given a ration of RUTF according to their weight (average 180–200 kcal/kg/day) and at the second visit anthelminthic treatment (albendazole) was given. The dosage for all medicines was age or weight related and given according to the Malawi National Guidelines.

Study subjects were requested to attend outpatient treatment for SAM on a weekly basis. At each clinic visit the child was measured for weight and MUAC and their appetite for RUTF was assessed. Due to the rotation of MoH clinical staff in attendance at the health centres, a single observer from the research team conducted each of the measurements of weight, height, and MUAC in order to minimise inter-observer variation. The standardisation of the measurements was assessed using the method of Habicht [9], using the principle investigator as the standard, and was reviewed every 3 months.

If the status of the child had not improved or had deteriorated since the previous visit then the child was referred to the MoH clinician for further assessment according to Malawi National Guidelines. Any child absent from treatment was followed up at home by a Health Surveillance Assistant (HSA) or community-based volunteer and encouraged to return for treatment.

Each study subject was discharged ‘cured’ from outpatient treatment when they achieved a MUAC of 125 mm or greater on two consecutive clinic visits. On discharge the caregiver was given a fourteen-day supply of RUTF calculated according to the weight of the child. The child was referred to a Supplementary Feeding Program (SFP) if such a program were operating in the health area of residence. A study subject was also discharged from the study if the child died, ‘defaulted’ (i.e. was absent for three consecutive visits) or was ‘non-cured’ (i.e. had not reached the discharge criteria) after sixteen weeks (112 days). All admissions and discharges from the study were approved and verified by a member of the Valid International research team. Any study subject discharged from the study as ‘non-cured’ was also assessed according to Malawi National Guidelines and referred back to the standard Ministry of Health programme for further treatment if the child had not reached the discharge criteria as ‘cured’ according to national guidelines.

Previous studies had indicated that most mortality following treatment for SAM occurs within three months of discharge [10]. Each child that was discharged as ‘cured’ was followed up every two weeks for a three-month period by an HSA trained in the study protocols. At each follow-up visit the child was assessed for illness (cough, fever, diarrhoea or vomiting) and their MUAC was measured by the HSA in accordance with their training in the Integrated Management of Childhood Illnesses (IMCI) program. Any child who had relapsed (i.e. met the program admission criteria of MUAC ≤ 115 mm or had bilateral pitting oedema) or had deteriorated clinically was referred back to the health centre for assessment and treatment. Relapsed children were eligible for re-admission to the study if their MUAC was equal to or below 115 mm. Any child absent from their home for two consecutive home visits was verified as being lost to follow-up (LTFU) by consulting with members of the local community regarding the whereabouts of the family. The accuracy of the MUAC measurements and clinical status of the child during follow-up was assessed by a member of the research team. Verification visits were made randomly to children in the follow-up phase of the study and visits were conducted in all cases where relapse was detected or LTFU had occurred.

No preferential treatment was given to study subjects compared with non-study subjects.

### Data collection and analysis

On admission and at each subsequent visit, the child’s weight was measured (naked or wearing light undergarments and without shoes) to the nearest 100 g using Salter™ scales (Salter-Brecknell 235-6S series), the model used by the health centre for the clinical management of SAM cases. MUAC was measured to the nearest millimetre using a standard non-elastic MUAC tape (UNICEF supply code S0145620 “MUAC, Child 11.5, Red/PAC-50”). On admission and discharge the child’s height was measured to the nearest millimetre using a standard paediatric height board (supine length was measured for children less than 24 months of age).

After discharge as ‘cured’, the child was followed up at home every two weeks for a three-month period by a HSA trained in the study protocols. At each follow-up visit the child was assessed for illness (cough, fever, diarrhoea or vomiting) and their MUAC was measured by the HSA.

Data were collected on paper forms designed for the purpose and were entered into an EpiData Version 3.1 (EpiData Association, Odense, Denmark) database using both interactive checking for range and legal values and double-entry validation. Statistical analysis was performed using the *R* Language for Data Analysis and Graphics [11]. Height-for-age, weight-for-age, and weight-for-height z-scores were calculated according to the WHO Child Growth Standard [12]. The Kruskal-Wallis non-parametric test was used for comparisons of lengths of stay and proportional weight gain by categories of MUAC at admission and categories of height at admission and for comparison of height at admission for ‘cured’ and ‘non-cured’ cases.. The *null hypothesis* for the Kruskal-Wallis test is that the data in each group have the same distribution of the variable of interest. The strength of association between cure and MUAC at admission and attendance rate was investigated using logistic regression.

## Results

Table 1 and 2 describes the study cohort at admission. The study recruited 258 children aged between 6 and 51 months between February 2011 and March 2012 of whom 115 (44.6 %) were male and 143 (55.4 %) were female. The study protocol included children aged up to 59 months but none were older than 51 months. Of the children enrolled in the study, 125 (48.4 %) did not present with any concurrent illnesses (defined as diarrhoea, vomiting, fever, or acute respiratory symptoms) while 27 (10.5 %) presented with complications severe enough to require transfer to inpatient care prior to enrolment in the study. The remaining 106 (41.1 %) of children had concurrent illnesses as outlined in Table 1 and 2. 140 (54.3 %) of children were tested for HIV on admission and of those children, 19 (13.6 %) of 140 (54.3 %) children were tested for HIV on admission tested positive for HIV.

**Table 1:** Main characteristics of study subjects at admission (*n =* 258^a^)

Characteristic	Number	Proportion
Males	115	44.6 %
Females	143	55.4 %
Number with MUAC ≤115 mm^b^ only	248	96.1 %
Number with MUAC ≤ 115 mm and oedema	10	3.9 %
Requiring inpatient care^c^	27	10.5 %
No concurrent illnesses recorded^d^	125	48.4 %
Diarrhoea^d^	56	21.7 %
Vomiting^d^	23	8.9 %
Fever^d^	65	25.2 %
Respiratory illness^d^	73	28.3 %
HIV positive^e,f^	19	7.4 %
HIV negative	121	46.9 %
HIV status not known (i.e. testing refused)	118	45.7 %

^a^There were no refusals

^b^MUAC tapes were marked in divisions of 1 mm and color-coded. A’red MUAC’, signifying severe acute malnutrition, was marked red at the 115 mm cut off point

^c^Inpatient stabilisation care prior to enrolment in the study as per CMAM National Guidelines

^d^Does not sum to 100 % due to some subjects having multiple illnesses at presentation

^e^Serial HIV testing according to national protocol using Determine™ and Uni-Gold Recombigen™ HIV assays

^f^Prevalence of HIV in those tested was 13.6 %

**Table 2:** Main characteristics of study subjects at admission (*n =* 258^a^)

Characteristic	Minimum	Lower Quartile	Median	Mean	Upper Quartile	Maximum
Age at admission (months)	6.0	10.0	14.0	16.4	21.0	51.0
MUAC at admission (cm)^c^	8.0	10.4	11.0	10.8	11.4	11.5
Height at admission (cm)	53.3	62.5	67.4	67.6	72.2	92.5
Median duration of inpatient episode (days)^b^	2.0	5.0	7.0	8.3	9.0	25.0

^a^There were no refusals

^b^Inpatient stabilisation care prior to enrolment in the study as per CMAM National Guidelines

^c^101 (39.1 %) of admissions were below 65 cm in height

**Fig. 1:**
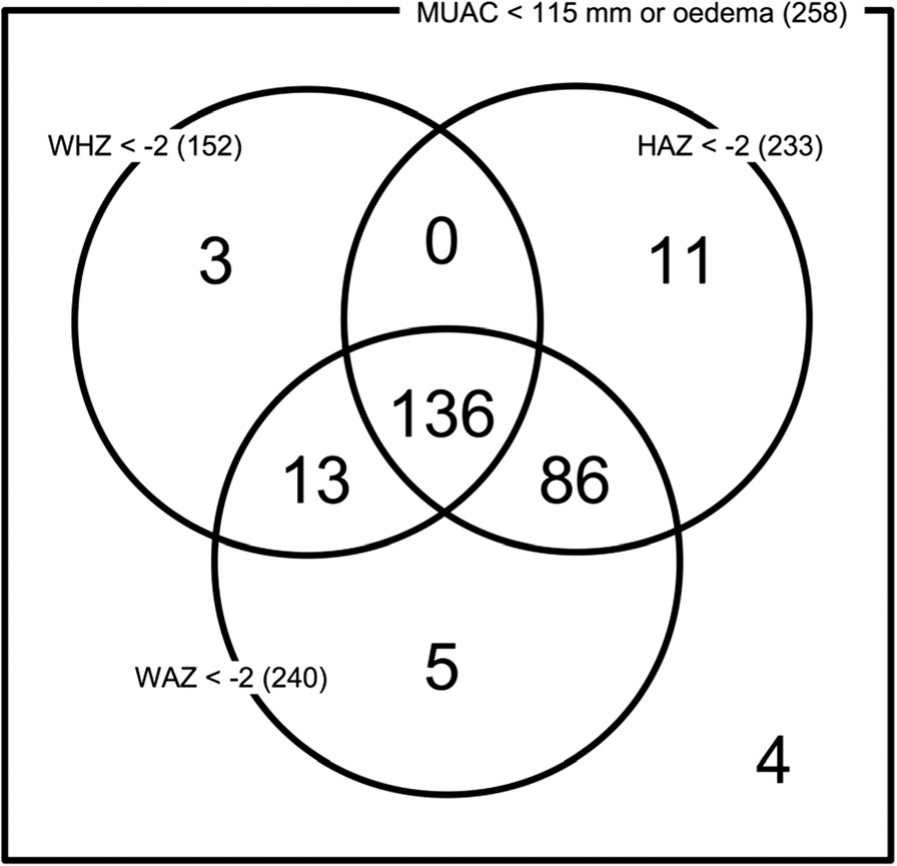
Venn diagram showing anthropometric categories of all admissions

Figure 1 summarises the anthropometric data of the study subjects at admission in a Venn diagram [13] in terms of MUAC, weight-for-height z-score (WHZ), height-for-age z-score (HAZ) and weight-for-age z-score (WAZ) according to the World Health Organization Growth Standards [12]. Only 4 (1.6 %) of the recruited subjects did not have a WHZ < −2, HAZ < −2, or WAZ < −2 on admission. The majority (52.9 %) of the study cohort was undernourished by all three indicators and by MUAC. A large majority (c. 90 %) of study subjects were stunted.

Table 3 describes the treatment given to the study subjects. A high proportion of subjects (93.0 %) received antibiotics while 65.0 % received the full national protocol of medicines.

**Table 3:** Treatment given in programme

Treatment	Number	Percentage
RUTF^a^	258	100.0 %
Vitamin A	203	78.7 %
Antimicrobials	238	92.2 %
Anthelminthics^b^	108	67.1 %
Antimalarials	82	31.8 %
Received full CMAM protocol^c^	168	65.1 %
Referred for inpatient care^d^	35	13.6 %

^a^Quantity given according to national protocol (average 180–200 kcal/kg/day)

^b^Given to eligible children ≥ 12 months only (according to national protocol). 79.1 % of all cases received the correct age appropriate intervention

^c^As per CMAM National Guidelines

^d^Children referred to inpatient care as per CMAM National Guidelines

**Fig. 2:**
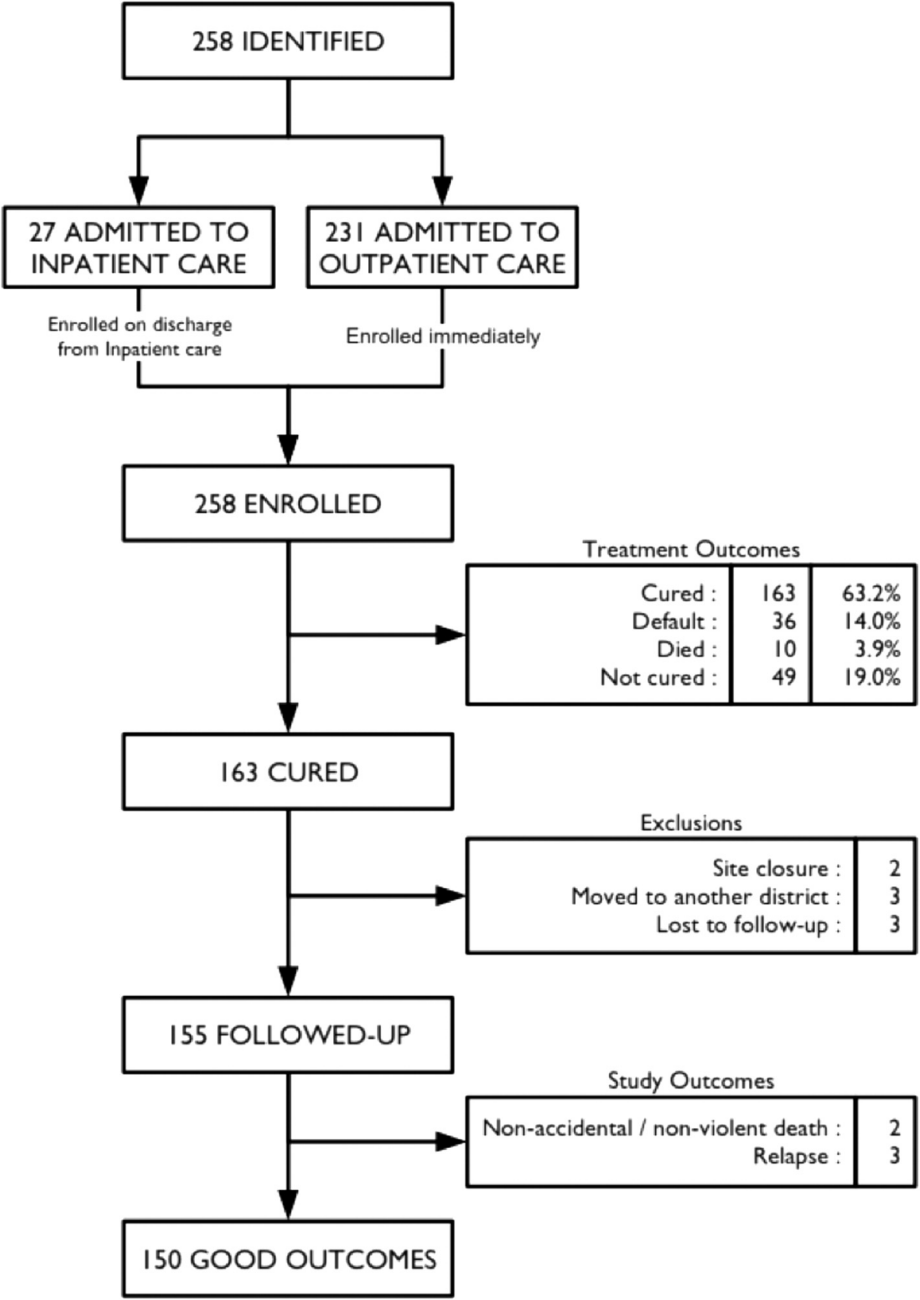
Flow diagram showing outcomes and exclusions. The study was discontinued at two health centres due to a fall to zero recruitment

Figure 2 describes the treatment outcomes achieved in the study and the subsequent exclusions from follow-up. 63.2 % of enrolled subjects were cured while 14.0 % defaulted, 3.9 % died during treatment and 19.0 % were discharged as ‘non-cured’. The cure rate for the study did not meet the Sphere Minimum Standard of 75 % [14], however, defaulter rates did meet Sphere minimum standards (i.e. less than 15 %) and the mortality rate was lower than and met the criteria for success set by WHO for community based programmes in non-emergency contexts (i.e. < 5 %) [7, 15]. The low cure rate observed was primarily due to children being discharged from the study after 4 months of treatment without attaining the study discharge criteria. Of the 49 cases discharged as ‘non-cured’ from this study 43 (87.8 %) were discharged as ‘cured’ according to the existing national guidelines (MUAC > 115 mm and 2 months minimum stay) while 6 (12.2 %) required continuing care.

**Fig. 3:**
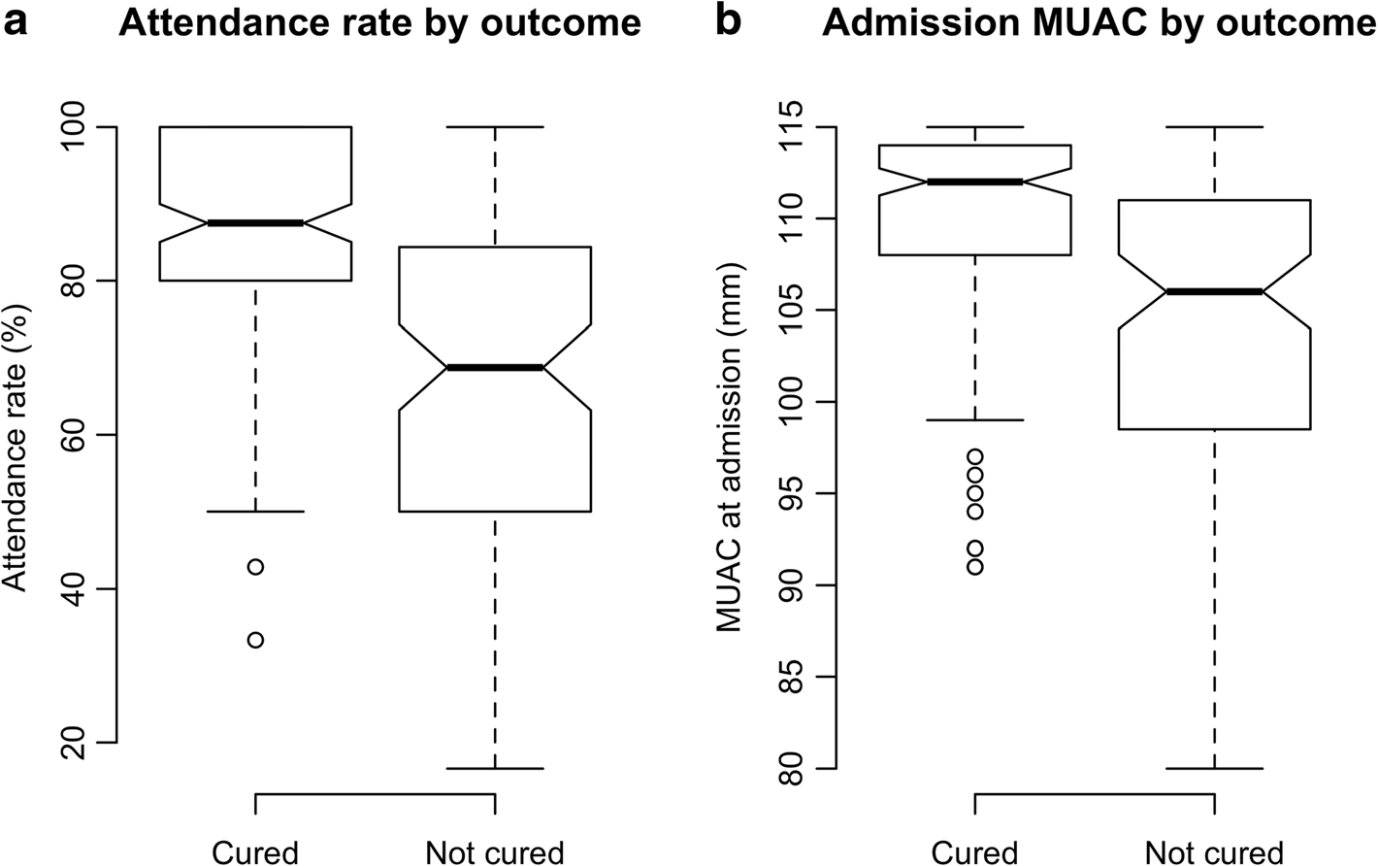
**a** Box plot describing Attendance rate for ‘cured’ and ‘non-cured’ SAM cases. **b**: Box plot describing MUAC at admission for ‘cured’ and ‘non-cured’ SAM cases

The median attendance rate for ‘cured’ patients (as a percentage of the total number of weekly visits required of all subjects by the study protocol during treatment) was 87.0 %. Figure 3a shows a box plot for the attendance rate for ‘cured’ and ‘non-cured’ children and Fig. 3b shows a box plot for the MUAC-at-admission for ‘cured’ and ‘non-cured’ children. Attendance and MUAC-at-admission variables were both independently associated with cure (*p <* 0.0001). Each millimetre increase in admission MUAC resulted in a 2.2 % (95 % CI = 1.47 %; 2.85 %) increase in the odds of cure. Each percentage point increase in the attendance rate was associated with a 1.13 % (95 % CI = 0.89 %; 1.38 %) increase in the odds of cure. These increases appear small but they are for small increases in variables with wide ranges. A ten millimetre increase in admission MUAC (e.g. from 102 mm to 112 mm) was associated with a 23.8 % (95 % CI = 15.7 %; 32.4 %) increase in the odds of cure. A twenty-percentage point increase in attendance (e.g. from 60 % to 80 %) was associated with a 25.2 % (95 % CI = 19.4 %; 31.5 %) increase in the odds of cure.

The 19 children identified as being HIV positive showed similar treatment outcomes to other children: ‘Cured’ 52.6 %, ‘Died’ 5.3 %, ‘Default’ 21.1 %, and ‘Non-cured’ 15.8 %.

**Fig. 4:**
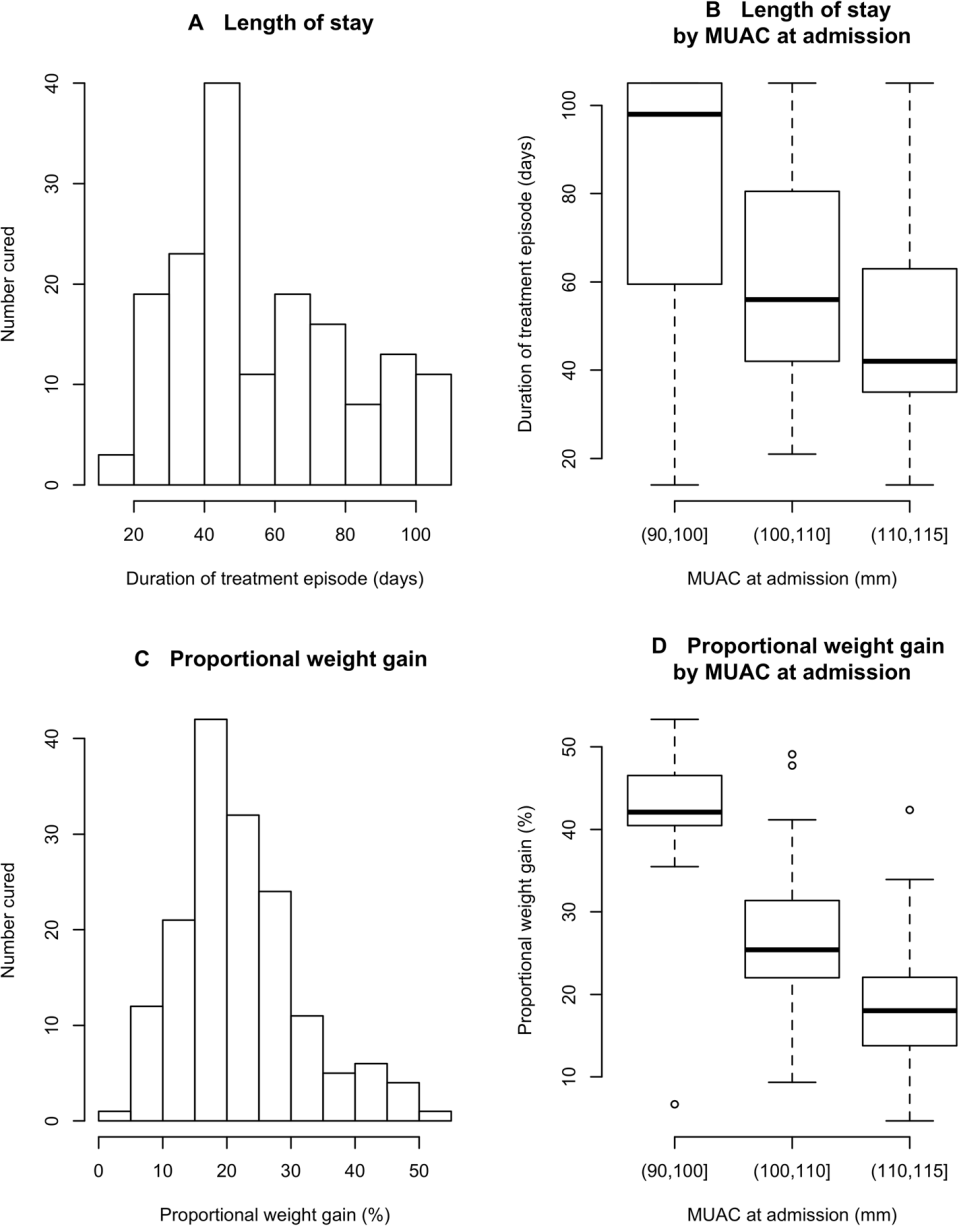
**a** Histogram showing distribution of duration of treatment episode. **b**: Box plots describing the length of stay for three classes of MUAC at admission **c** Histogram showing the distribution of proportional weight gains at discharge. **d** Box plots describing proportional weight gain at discharge for three classes of MUAC at admission

Figure 4a shows the distribution of duration of treatment episode while Fig. 4b presents box plots describing the length of stay for three classes of MUAC at admission. Figure 4c shows the distribution of proportional weight gains. Figure 4d presents box plots describing proportional weight gain for three classes of MUAC at admission. The median length of stay of all children in the study was 49 days (IQR = 35 days—77 days). Children identified as HIV positive and discharged ‘cured’ had a median duration of treatment of 84 days (IQR = 63–105 days). Children with lower MUAC at admission required longer durations of treatment (Kruskal-Wallis test, *p <* 0.0001) with a median duration of treatment in the lowest MUAC group of 98 days (IQR = 60 days – 105 days) and in the highest MUAC group of 42 days (IQR = 35 days—63 days).

The median proportional weight gain of all children in the study was 21 % (IQR = 16 % − 27 %). Children with lower MUACs experienced higher proportional weight gains (Kruskal-Wallis test, *p <* 0.0001) with median proportional weight gain of 42 % in the lowest MUAC group (IQR = 40 % − 47 %) and 18 % in the highest MUAC group (IQR = 14 % − 22 %).

**Fig. 5:**
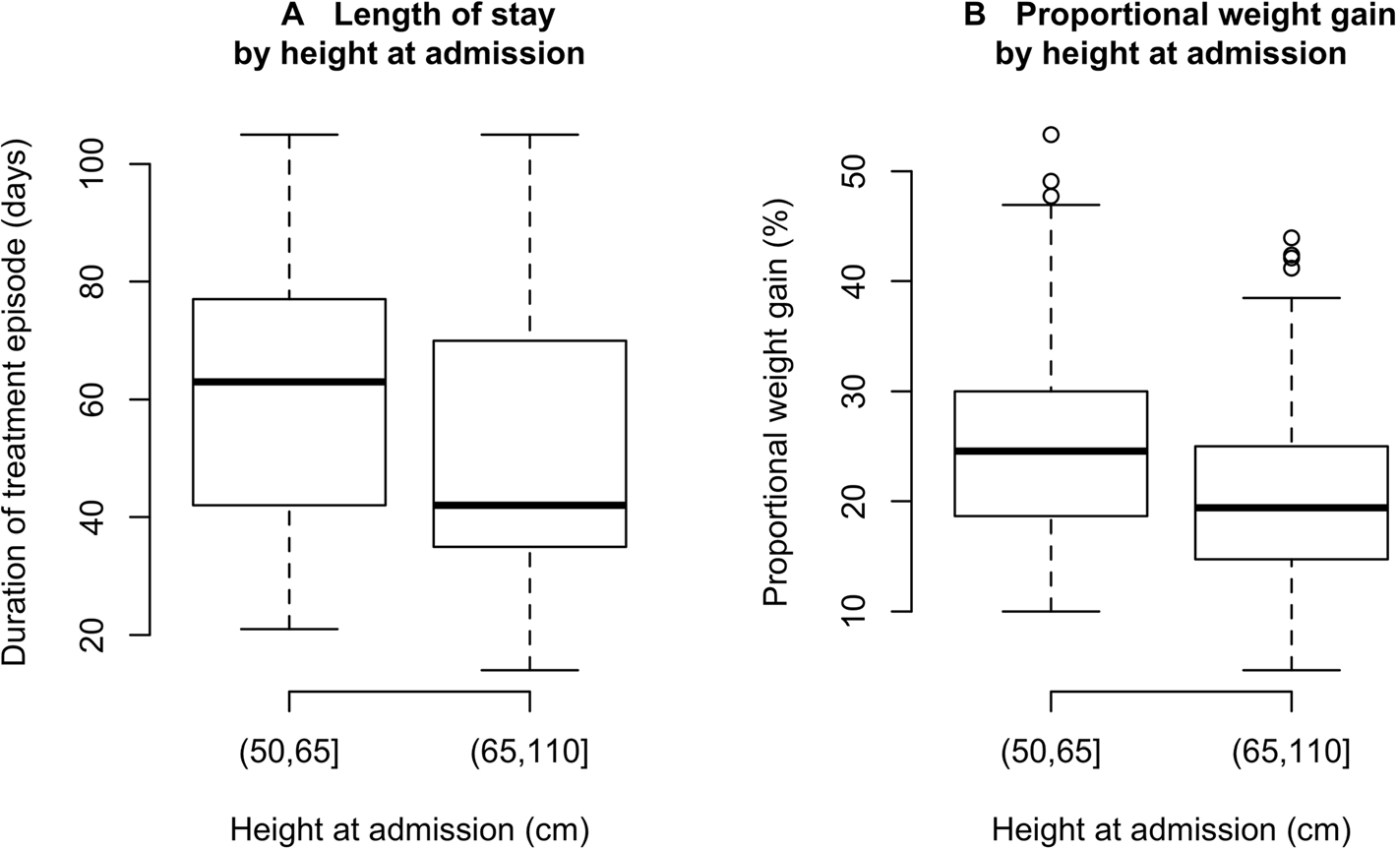
**a** Box plot describing length of stay by height at admission for two height classes. **b**: Box plot describing proportional weight gain by height at admission for two height classes

Figures 5a and b present box plots describing the length of stay and proportional weight gain by height at admission for two height classes at admission respectively. Children recruited to the study with a height at admission below 65 cm showed a higher proportional weight gain (Kruskal-Wallis test, *p =* 0.0003) and a longer median length of stay under treatment than taller children (Kruskal-Wallis test, *p =* 0.0145). These children were no more likely to be discharged as ‘non-cured’ than taller children (relative risk (RR) = 0.93, 95 % CI = 0.77 − 1.13, chi-square = 0.33, *p =* 0.5667). There was no association with negative outcomes at three months post discharge for children with heights at admission below 65 cm than for taller children (RR = 1.22, 95 % CI = 0.21–7.09, Fisher’s exact test (single-tailed with H_A_ := RR > 1), *p =* 0.5798).

The median height at admission of ‘cured’ cases was 67.5 cm (IQR = 63.5–72.6) and the median height at admission of ‘non-cured’ cases was 66.5 cm (IQR = 60.8–70.1). Lower height at admission was not significantly associated with being’non-cured’ (Kruskal Wallis test, *p =* 0.0645).

The median height at admission for those known to have died was 62.2 cm (IQR = 60.1–65.9) and the median height at admission for those not known to have died was 67.5 cm (IQR = 63.0–72.3). Lower height at admission was significantly associated with mortality (Kruskal Wallis test, *p =* 0.0369). This analysis should be interpreted with caution as defaulter follow-up was limited and deaths in defaulters may have not been detected and recorded.

Eight children were excluded from the follow-up portion of the analysis. Follow-up was discontinued in these cases due to closure of the study site, relocation of the subject to another district, and inability to be followed because the family were from outside of the health district and had provided an incorrect address upon registration in order to qualify to receive care.

The proportion of patients discharged as ‘cured’ and experiencing relapse to SAM (1.9 %, 95 % CI = 0.4 %–5.6 %) or non-violent/non-accidental death (1.3 %, 95 % CI = 0.2 %–4.6 %) was 3.2 % (95 % CI = 1.1 %–7.4 %, *p =* 0.0013, binomial test with the alternative hypothesis that the proportion of failures is below 10 %). None of the children identified as HIV positive and discharged ‘cured’ either ‘relapsed’ or ‘died’ within the three-month follow-up period.

Of the 155 children followed up after being discharged as ‘cured’, 29 % (*n* = 45) receivedSFP rations. There were no negative outcomes amongst these 45 children after three months of follow-up (Risk difference = 4.55 %, 95 % CI = 0.65 %–8.44 %, Fisher one-tailed p-value = 0.1752). Of the 45 children receiving SFP, 20 % (*n* = 9) became moderately acutely malnourished (MAM) with a MUAC between 11.5 and 12.4 cm during the follow-up period. 44 % (*n* = 4) of those who became MAM during follow-up remained MAM at the last follow-up visit.

The study data for the children who ‘died’ or ‘relapsed’ are shown in Table 4. A detailed follow-up investigation of the deaths indicated that in both cases the child had accessed medical services at a hospital prior to death.

**Table 4:** Details of study subjects with negative outcomes in the three months following their discharge with a MUAC of 125 mm or greater (*n* = 5)

		Admission		Discharge				Follow-up		
Outcome	Sex	Age (months)	MUAC (mm)^a^	MUAC (mm)	WHZ	Length of stay (days)	Weight gain (%)	Duration of follow-up^b^ (weeks)	Last known MUAC (mm)^c^	Illness during follow-up
Death	F	9	112	125	+0.30	56	10.0	10	134	diarrhoea cough
Death	F	14	110	128	−0.01	105	14.7	6	120	diarrhoea vomiting fever cough
Relapse	M	14	110	126	−0.08	84	26.8	12	110	none recorded
Relapse	M	12	104	130	−0.39	35	26.8	8	114	diarrhoea vomiting fever cough
Relapse	F	9	110	128	−0.65	112	30.0	6	115	diarrhoea

^a^None of the children followed-up had oedema at admission

^b^The follow-up visit prior to death or the follow-up visit when relapse was detected

^c^Last known MUAC for deaths is the MUAC recorded in the follow-up visit prior to death. Last known MUAC for relapsed cases is the MUAC at the follow-up visit at which relapse was detected. Relapsed cases were referred back to the therapeutic feeding program

Regarding relapses, one child was absent for 4 out of the 6 follow-up visits prior to relapse but did not have any illnesses recorded; the second child was recorded to have had diarrhoea, fever and cough in the week prior to relapse and the third child had a cough recorded for weeks 2 and 3 but no other illnesses recorded during the week prior to the relapse being detected in week 5.

### Limitations

There are several limitations to this study. Occasional interruptions in the MoH supply of routine medicines lead to shortages of some medicines. When this occurred the MoH clinician decided on the appropriate use of resources; no priority was given to study subjects. The *National Health Sciences Research Committee of Malawi* was consulted and ethical approval was given to continue the study despite not all of the study subjects receiving the full CMAM protocol of treatment. The results thus reflect the outcomes achieved in a MoH treatment setting with limited resources.

Following discharge from the study cohort as ‘non-cured’ according to the study criteria, children were assessed for the need for continued treatment or discharged according to the Malawi National guidelines. The compliance and outcome of children who were discharged from the study cohort but who continued treatment or were discharged as ‘cured’ were not followed up as part of this study.

The limited follow-up of absentees and defaulters during the course of treatment contributed to a reduction in the proportion of the children who were recruited to the study and later discharged as ‘cured’, thus limiting the sample size of the study.

The study did not follow compliance with referral for antiretroviral prophylaxis or treatment for HIV for HIV positive cases, however only small differences were observed in programmatic outcomes between HIV positive and HIV negative subjects. The small number of HIV positive subjects did not allow disaggregation of the duration of treatment by MUAC on admission. This issue might be investigated in a larger cohort of HIV positive subjects receiving nutritional rehabilitation prior to starting antiretroviral therapy.

The analyses carried out with this study design were not able to demonstrate that alternative discharge criteria would have resulted in fewer negative outcomes than a discharge criterion of MUAC ≥ 125 mm.

## Discussion

The study confirms that a discharge criterion of MUAC greater than 125 mm for two consecutive visits represents a practicable and safe discharge criterion according to the standard established for the study and is also an appropriate discharge criterion for children with a height of less than 65 cm at admission. Relapses formed 1.9 % (95 % CI = 0.4 %–5.6 %) of the cohort of ‘cured’ patients and non-accidental and non-violent deaths formed 1.3 % (95 % CI = 0.2 %–4.6 %) of the cohort of ‘cured’ patients over the three-month follow-up period. Negative outcomes occurred in 3.2 % (95 % CI = 1.1 %–7.4 %) of the cohort of ‘cured’ patients over the three-month follow-up period. None of the negative outcomes observed during the 3-month follow-up were in children identified as HIV positive at admission. Post-discharge deaths were not associated with relapse to SAM and were not associated with having a discharge WHZ below −2 z-scores. While there are no internationally agreed standards for relapse and mortality following cure from CMAM programmes, all cases with a negative outcome following discharge had a discharge WHZ above −1 z-scores. This suggests that MUAC ≥ 125 mm for two consecutive visits is as at least safe as discharge using WHZ > − 1 z-scores. The rate of relapse and mortality following discharge as ‘cured’ reported in this study are also similar or lower to those reported by Ashworth in other studies using weight for height (e.g. WHZ > −1 or weight-for-height percentage of median > 85 %) as the discharge criterion from CMAM programmes [7].

Children with lower MUAC at admission had longer durations of treatment and higher proportional weight gains than the children admitted with a higher MUAC. This shows that the MUAC discharge criterion ensures children who are the most malnourished remain under treatment longer and are discharged with a higher proportional weight gain. This eliminates the problems previously identified with the proportional weight gain criterion [4] and its association with under-treatment for the most malnourished individuals. Children identified as HIV positive at admission had a longer median duration of treatment than that observed for all children combined.

Children with height less than 65 cm at admission also showed greater proportional weight gain and had longer lengths of stay than taller children and showed no association with negative outcomes three months after discharge. These findings may be partly explained due to younger children typically having higher weight gains even when well nourished and that, on average, younger children are shorter. However, these children respond and benefit the most from treatment, and the evidence of this study should allay concerns that using MUAC is not appropriate as an admission or discharge criterion for stunted children aged 6 months or older with a height below 65 cm.

A high proportion of children (92.2 %) received antimicrobials during treatment although the compliance with protocol was lower for Vitamin A and anthelminthics. A low proportion of children (65.1 %) received the full CMAM protocol of medicines. This was due to shortages of supply at the clinics. Whilst this may represent a limitation to this study, the outcomes of the study reflect the context of an MoH programme with supply shortages.

Reasons for defaulting included waiting times for treatment at the clinic site and prioritisation of household labour (e.g. harvesting). Some defaulters had reportedly migrated away from their temporary home in the treatment catchment area.

It was expected that enrolment into SFP after cure would have a large positive effect on negative outcomes following discharge, however, negative outcomes were rare and any analysis will lack statistical power to detect all but very large effects. The data suggest a small positive effect of SFP enrolment may be present.

Study findings included a high non-cure rate (18.9 %) for all children combined (15.8 % for children identified as HIV positive at admission). MUAC at admission and attendance rate were positively associated with cure.

In the absence of the incentivised follow-up of absentees and defaulters; reducing waiting and transit times for clinic visits, frequent counselling to refrain from sharing RUTF, and case review by physicians did little to improve attendance and reduce the non-cure rate over time.

These results provide strong evidence that the proportion ‘cured’ can be influenced by program design and activities that improve the detection, early recruitment and retention of SAM cases as well as compliance to the SAM treatment protocol.

Care should be taken in extrapolating these results to other populations, however data from CMAM programmes using the same discharge criterion in other contexts report similar findings in terms of median duration of treatment (approximately 8 weeks or 56 days). [3, 4].

Additional studies may be needed to assess whether a discharge criterion of MUAC ≥ 125 mm is safe in other CMAM programs with low levels of supervision and in other settings. There are currently no internationally agreed standards by which to assess the safety of MUAC or other discharge criteria, however this study indicates that low rates of relapse and mortality following cure are achievable in low resource, non-emergency settings.

## Conclusions

This study confirms that a discharge criterion of MUAC ≥ 125 mm is safe and practical leading to low mortality and relapse at three months post discharge in the context of Malawian Ministry of Health outpatient care facilities. Negative outcomes were not associated with failing to meet the WHZ > −2 z-scores criterion. This MUAC discharge criterion was also found to be appropriate for children aged six months or older who are less than 65 cm in height at admission. Compliance, as measured by attendance rate, and MUAC at admission were both positively associated with cure.

Children admitted with lower MUAC remain under treatment longer and achieve higher proportional weight gains when MUAC discharge criteria are used. The introduction of MUAC discharge may lead to longer average length of stay initially where there is limited case finding and admission MUAC may be expected to be low. Early detection and recruitment of severe acute malnutrition cases using MUAC in the community and compliance with the CMAM treatment protocols should, however, reduce lengths of stay and associated treatment costs.

## Abbreviations

AED: academy for educational development
CI: confidence interval
CMAM: community based management of acute malnutrition
FANTA: food and nutritional technical assistance
FHI: family health international
HIV: human immunodeficiency virus
HSA: health surveillance assistant
LTFU: lost to follow-up
MoH: ministry of health
MUAC: mid upper arm circumference
NGO: non governmental organisation
NHSRC: national health sciences research committee
NRU: nutritional rehabilitation unit
SAM: severe acute malnutrition
UN: United Nations
UNICEF: United Nations children’s fund
UNSCN: United Nations standing committee on Nutrition
WFP: world food programme
WHO: world health organisation
WHZ: weight for height z score
